# Osteogenic mechanism of chlorogenic acid and its application in clinical practice

**DOI:** 10.3389/fphar.2024.1396354

**Published:** 2024-05-30

**Authors:** Jiayu Shen, Shichen Zhang, Jiayu Zhang, Xin Wei, Zilin Wang, Bing Han

**Affiliations:** ^1^ Department of Oral and Maxillofacial Surgery, Hospital of Stomatology, Jilin University, Changchun, China; ^2^ Jilin Provincial Key Laboratory of Tooth Development and Bone Remodeling, Jilin University, Changchun, China

**Keywords:** chlorogenic acid, osteoblast, osteoclast, osteoporosis, periodontitis

## Abstract

Natural polyphenols may have a role in counteracting oxidative stress, which is associated with aging and several bone-related diseases. Chlorogenic acid (CGA) is a naturally occurring polyphenolic compound formed by the esterification of caffeic and quininic acids with osteogenic, antioxidant, and anti-inflammatory properties. This review discusses the potential of CGA to enhance osteogenesis by increasing the osteogenic capacity of mesenchymal stem cells (MSCs), osteoblast survival, proliferation, differentiation, and mineralization, as well as its ability to attenuate osteoclastogenesis by enhancing osteoclast apoptosis and impeding osteoclast regeneration. CGA can be involved in bone remodeling by acting directly on pro-osteoclasts/osteoblasts or indirectly on osteoclasts by activating the nuclear factor kB (RANK)/RANK ligand (RANKL)/acting osteoprotegerin (OPG) system. Finally, we provide perspectives for using CGA to treat bone diseases.

## 1 Introduction

Bones are a very specialized and dynamic organ that undergoes constant regeneration. Bone generation begins in fetal life, and regeneration continues after skeletal maturation (Siddiqui and Partridge, 2016). Bone homeostasis is maintained by osteoclastic bone resorption and osteoblastic bone formation within the basic multicellular unit, both of which are well coordinated in time and space to maintain bone integrity (Borciani et al., 2020; Kim et al., 2020). The dysregulation of bone homeostasis is associated with the development of jaw disorders (Freeman et al., 2021). MSCs can differentiate into multiple cell types, including adipocytes, myocytes, chondrocytes, and osteoblasts, under the influence of regulatory transcription factors. Osteoblasts produce osteoid by synthesizing and secreting type I collagen, which promotes their mineralization (Katsimbri, 2017; Na et al., 2021). Osteoclasts develop from hematopoietic stem cells in the bone marrow, mature in response to differentiation factors, such as macrophage colony-stimulating factor and receptor-activated nuclear factor kappa B (RANKL), and have the ability to absorb bone matrix. Mediators that inhibit osteoclast differentiation and their ability to resorb bone can lead to increased bone mass. Therefore, understanding the mechanisms of osteoclast bone resorption associated with jaw disorders is important (Yahara et al., 2020; Freeman et al., 2021; Udagawa et al., 2021).

**FIGURE 1:**
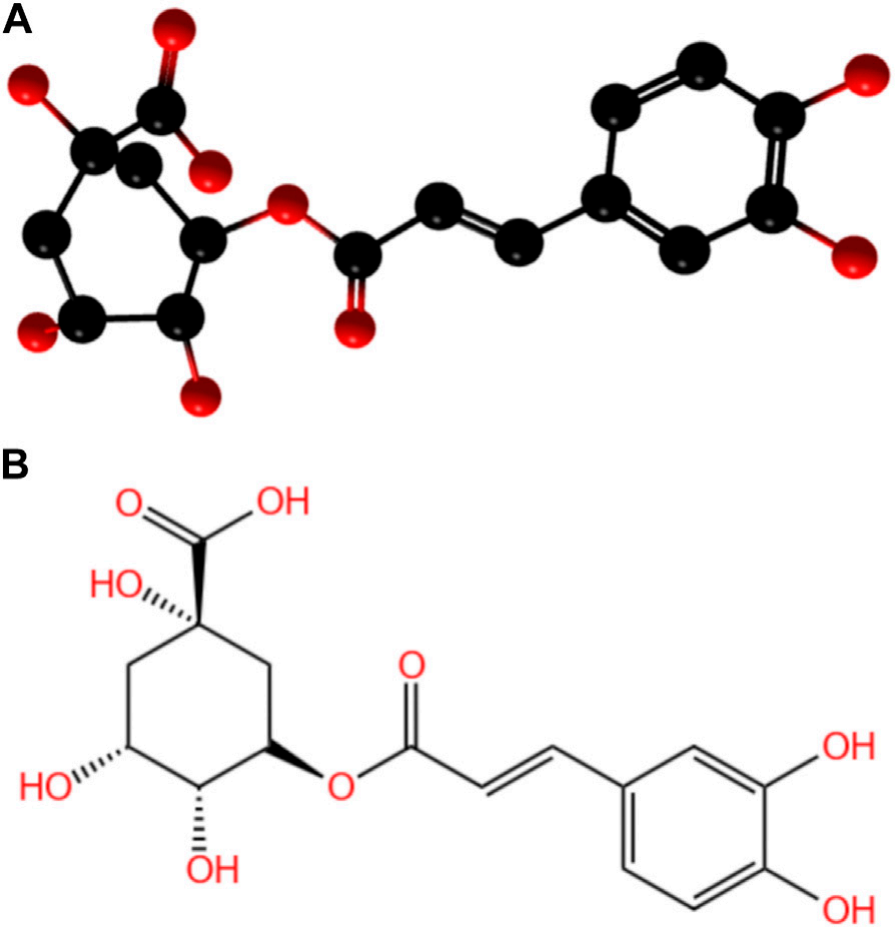
**(A)** 3D structure of chlorogenic acid; **(B)** Molecular formula of CGA.

With the development of herbal medicine, natural drug extracts have played increasingly important drug roles due to their efficiency and safety. Some researchers found that CGA could promote osteoblast activity and inhibit osteoclast activity (Kawabata et al., 2018; Wu et al., 2023). CGA is a polyphenolic compound formed by the esterification of caffeic acid and quininic acid and is known as 5-O-caffeoylquinic acid (Stefanello et al., 2019; Lu et al., 2020). (Figure 1)

**FIGURE 2:**
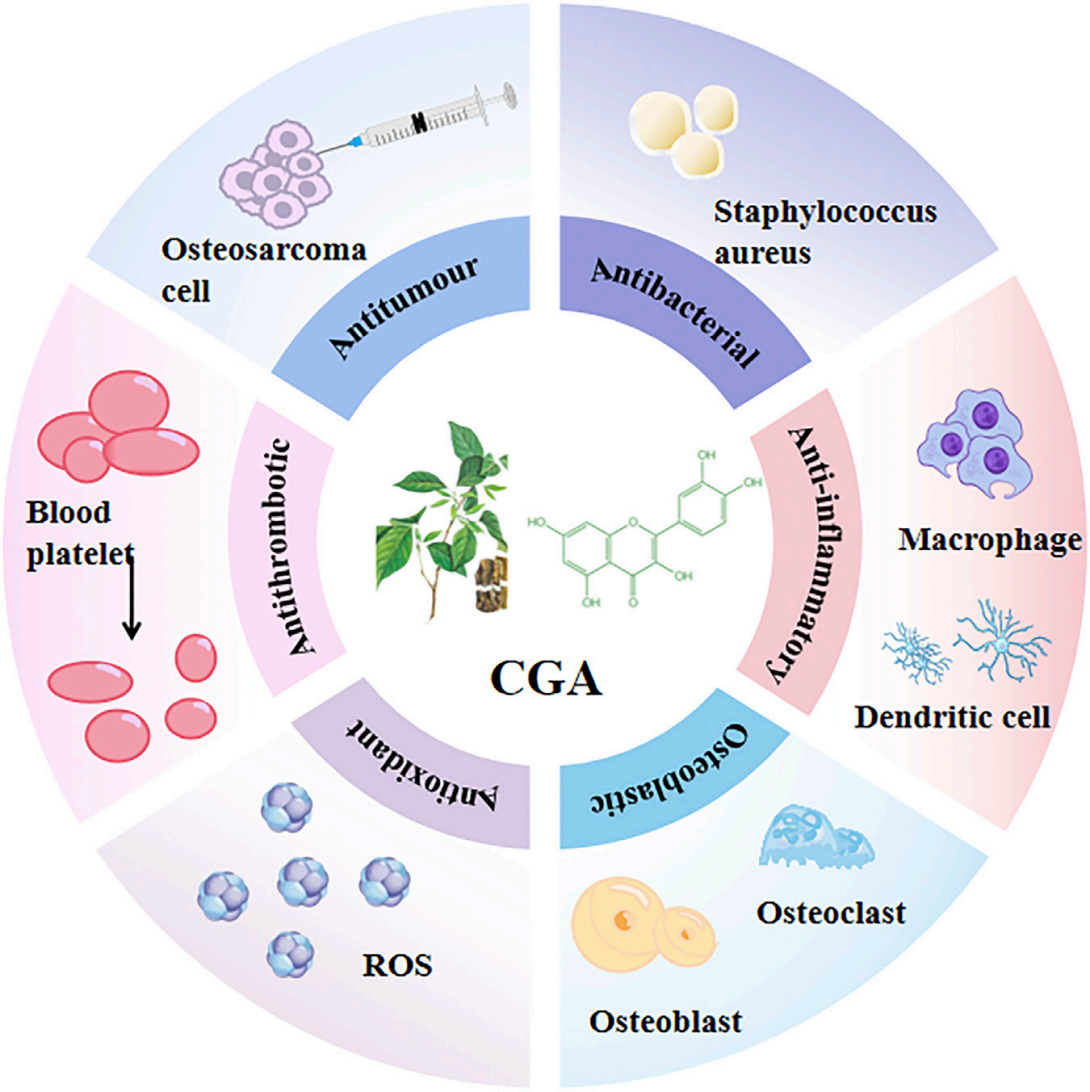
Pharmacological effects of CGA CGA has a variety of different pharmacological effects, such as antioxidant activity (Khochapong et al., 2021), antiviral, anti-inflammatory, antibacterial (Munteanu and Apetrei, 2021), antithrombotic, and antitumor activities (Ye et al., 2020).

CGA is not only the main active ingredient in many traditional Chinese medicines but also widely found in a variety of plant tissues and foods, such as coffee, beans, potatoes, plums, and honeysuckle. Previous reports showed that CGA exerts many different pharmacological effects, such as antioxidant (Khochapong et al., 2021), antiviral, anti-inflammatory, antibacterial (Munteanu and Apetrei, 2021), antithrombotic, and antitumor activity (Ye et al., 2020). (Figure 2) Clinical trials have also reported the beneficial effects of CGA on diabetes (Zhao et al., 2020; Mansour et al., 2021), hyperlipidemia (Ontawong et al., 2019; Ye et al., 2022), renal diseases (Zeng et al., 2022), and neurological disorders (Heitman and Ingram, 2017). (Table 1)

**TABLE 1:** Summary of potential medicinal benefits of chlorogenic acid.

Function	Chlorogenic acid-chitosan	Experimental models	**Mechanism of action**
**antioxidant activity**	Chlorogenic acid-chitosan	Rat pheochromocytomaline cell	CGA acts as an antioxidant through free radical and super radical scavenging and lipid peroxidation inhibition (Khochapong et al., 2021)
	Chlorogenic acid-rich foods and supplements	Menhaden oil was used as model lipid	CGA and its esters inhibit lipid oxidation by a combination of firee radical scavenging in the lipid phase and metal chelation in the aqueous phase (Santana-Gálvez et al., 2017)
	Caffeoyl Quinic Acid	None	CGA significantly inhibited oxidative stress-induced secretion of IL-8 and mRNA expression (Naveed et al., 2018)
	CGA	MC3T3-E1 cells exposed to Dex	CGA not only reversed the downregulation of p21, but also promoted the expression of nuclear Nrf2 and total Nrf2 and its downstream target protein HO-1, thus exerting antioxidant effects (Han et al., 2019)
**ant-inflammatory**	Curcumin and CGA	Rat vascular smooth muscle cells stimulated with LPS (L μg/mL)	Curcumin and CGA together reduced the mRNA expression of pro-inflammatory cytokines TNF-α, IL-6 and COX-2, possibly by suppression of NF-κβ, IκB-β-kinase and TLR-4 receptor at the mRNA level (Bisht et al., 2020)
	CGA mixed with vaseline	Skin inflammation in ears of ICR mouse induced by living Propionibacterium acnes	CGA treatment effectively rescued ear swelling, redness and erythema skin in ears of ICR mouse induced by P.acnes and significantly downregulated the expression of inflammatory cytokines by reducing the activity of the NF-κβ signalling pathway (Luo et al., 2021)
	1. CGA 2.Caffeic acid	1.Animal Models of Sepsisz	CGA’s anti-inflammatory effects involve inhibition of: NF-κβ, TNF-α, IL-1β, IL-6, PGE2 and JNK/AP-1 signaling pathway activation. Further, CGA inhibits the synthesis of other mediators, such as interferon-γ, monocyte chemotactic protein-l, and macrophage inflammatory protein-lα (Bagdas et al., 2020)
	3.Sinapic acid	2.Neurodegenerative Inflammatory Disease Models	
	CGA	Macrophages in obese mice	CGA in the peripheral synthesis/release of inflammatory mediators involved in these responses, such as TNFα and NO (dos Santos et al., 2006)
**antibacterial**	CGA	*S. pvogenes*	CGA may exert its antibacterial action through several actions, such as downregulating ribosomal subunits, affecting lipid metabolism, and scavenging intracellular ROS (Le et al., 2022)
	CGA loaded magnetic nanoparticles	Lung infection in 40 male Swiss albinomice	CGA acts by mainly disnupting the cell membrane of the microorganism, leading to its death (Shahabadi et al., 2023)
	CGA	The strains *Staphylococcus* aureusStreptococcuspnetoniae and *Escherichia coli*	CGA killed the bacteria by provoking irreversible permeability changes in the cell membrane, causing disruption of the membrane potential and loss of cytoplasm macromolecules including nucleotides (Lou et al., 2011)
**antitumour activities**	CGA	Glioma cells	CGA treatment influenced multiple cancer related pathways involving NF-κβ signaling, TGF-β signaling, MAPK signaling and TNF signaling pathways (Huang et al., 2019)
	CGA	A498 human kidney cancer cells	CGA inhibits proliferation and induces apoptosis in A498 human kidney cancer cells via inactivating PI3K/Akt/mTOR signalling pathway (Wang et al., 2019)

CGA, Chlorogenic acid; IL-8, Interleukin-8; MC3T3-El, The pre-osteoblast cell line; Dex, Dexamethasone; Nrf2, Nuclear factor erythroid 2-related factor 2; LPS, lipopolysaccharide; TNF-α, Tumor necrosis factor-α; COX-2, Cyclooxygenase 2; IL-6, Interleukin-6; PGE2, ProstaglandinE2; ROS, Reactive oxygen species; TGF-β, Transforming growth factor-β

**FIGURE 3:**
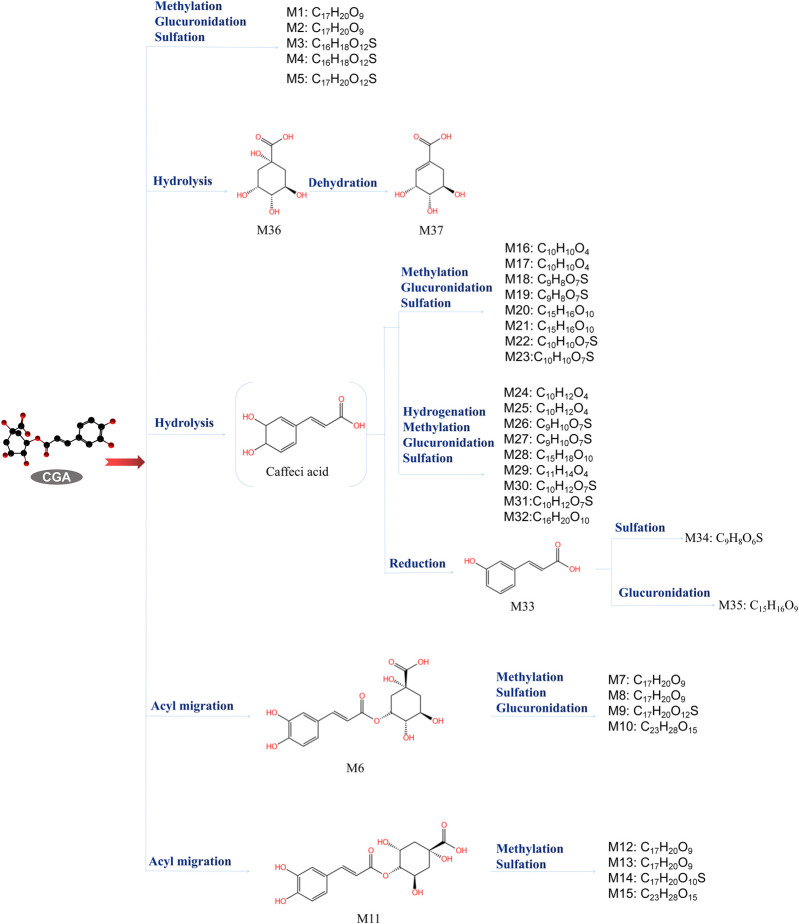
Chlorogenic acid metabolites (Zeng et al., 2022). The metabolic process of CGA and the structure and molecular formula of 37 metabolites.

**FIGURE 4:**
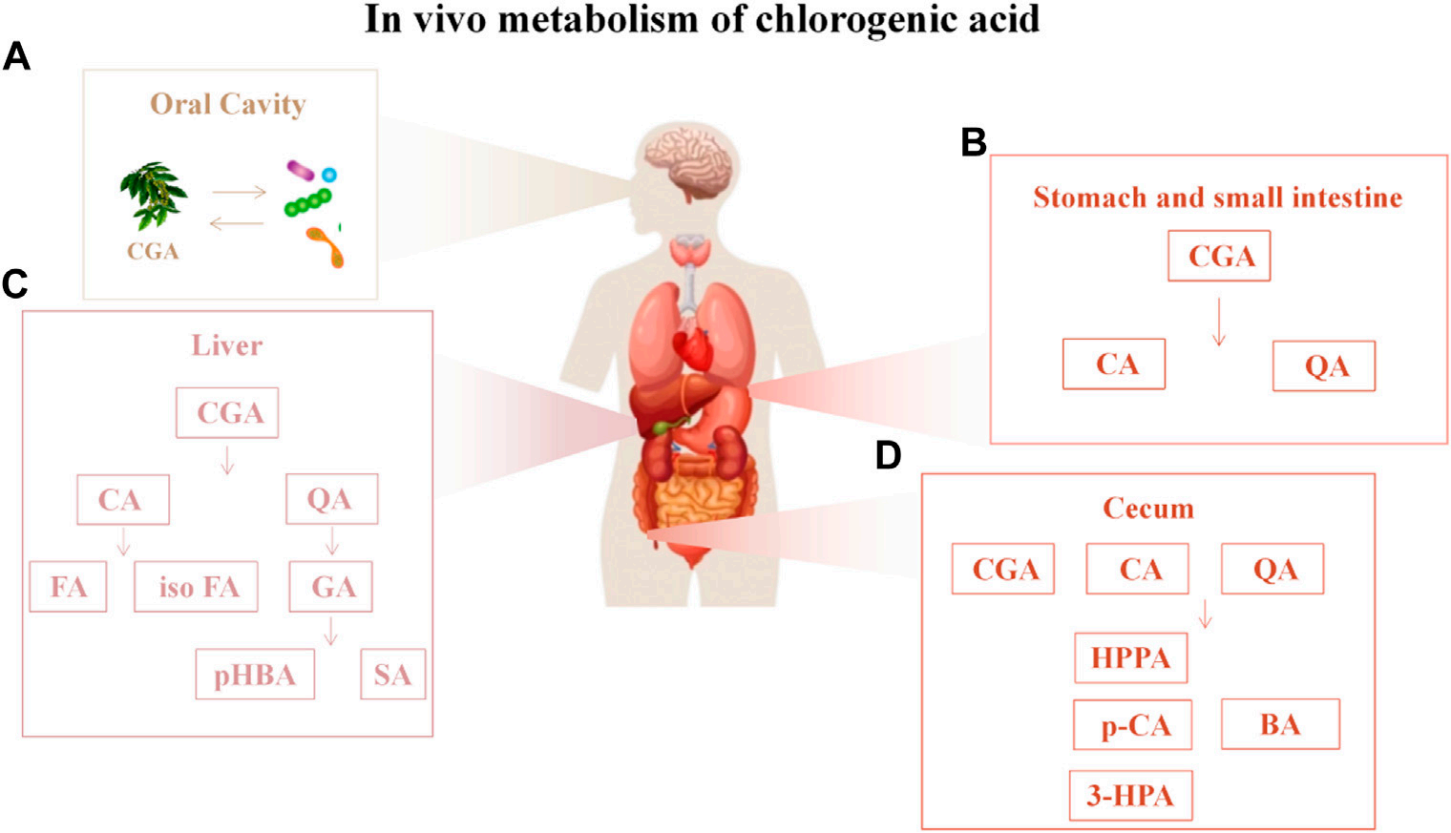
*In vivo* metabolism of chlorogenic acid **(A)** CGA is scarcely metabolized but can interact with the oral microbiota; **(B)** In the stomach and small intestine, chlorogenic acid (CGA) is metabolized into caffeic acid and quinic acid; **(C)** Upon entering the liver, caffeic acid is metabolized into ferulic acid and isoferulic acid, while quinic acid is converted into gallic acid, which is further degraded into p-hydroxybenzoic acid and syringic acid; **(D)** After entering the cecum in the form of CA and QA, CA is hydrolyzed into p-coumaric acid, 3-HPA or HPPA, and QA is hydrolyzed into BA. Abbreviations: CA, caffeic acid; QA, quinic acid; FA, ferulic acid; isoFA, isoferulic acid; GA, gallic acid; pHBA, 4-Hydroxybenzoic acid; SA, Gallic acid; HPPA, hydroxyphenyl propionic acid; p-CA, p-Coumaric acid; 3-HPA, 3-hydroxyphenyl propionic acid; BA, Benzoic acid.

CGA bioavailability is influenced by many factors, including oral and gastrointestinal conditions, hepatic-mediated metabolic processes (phase I and II metabolism), and gut and microbial flora (Alqahtani et al., 2021). In the oral cavity, CGA is scarcely metabolized but can interact with the oral microbiota, affecting its composition and metabolism, potentially playing a positive role in the prevention and treatment of oral diseases (Takahama et al., 2007; Barbour et al., 2022). A small fraction of CGA is absorbed intact in the stomach and small intestine, while the majority is metabolized into caffeic acid and quinic acid. These undergo further methylation, sulfation, and glucuronidation under the control of specific enzymes. Upon entering the liver, caffeic acid is metabolized to ferulic acid and isoferulic acid, while quinic acid is converted to gallic acid, which is further degraded to p-hydroxybenzoic acid and syringic acid. About two-thirds of CGA, in the form of CA and QA, enter the cecum, where it is hydrolyzed to p-coumaric acid, 3-(3-hydroxyphenyl) propionic acid, or 3-(3-hydroxyphenyl) acetic acid by the gut microbiota (such as *Escherichia coli*, *Bifidobacterium*, *Lactobacillus*, and *Enterococcus*) (Olthof et al., 2001; Lu et al., 2020). (Figure 3) Tomas-Barberan *et al.* suggested that the bioavailability of CGA metabolites largely depends on the composition and activity of the microbial community. By comparing the CGA-biotransforming capabilities of different human colonic microbiota, researchers found that diverse microbial communities act on CGA through different metabolic pathways, ultimately producing 3-(3-hydroxyphenyl) propionic acid (HPPA). Only a minority of individuals can further convert HPPA to phenylpropionic acid. Finally, the metabolites are absorbed and undergo phase II metabolism (conjugation with glucuronic acid, sulfate, methyl, or glycine) or other metabolic processes, such as hydrogenation, dehydrogenation, and α- or β-oxidation (Tomas-Barberan et al., 2014). Ultimately, 37 metabolites of CGA have been identified in human blood, urine, and feces (Nabavi et al., 2017; Lu et al., 2020; Liu et al., 2022). (Figure 4) This review discusses the potential mechanisms of CGA to regenerate bone tissue by promoting osteoblast activity and inhibiting osteoclast activity. Finally, we provide perspectives on the treatment of bone diseases with CGA.

## 2 Chlorogenic acid promotes osteoblasts

### 2.1 CGA enhances the proliferation and differentiation of osteoblasts

CGA has been shown to promote osteogenic proliferation and differentiation *in vivo*, thereby increasing bone mineralization and improving bone strength (Kawabata et al., 2018; Léotoing et al., 2016; Léotoing et al., 2016). These effects may be related to the amplification of tumor necrosis factor-α (TNF-α)-stimulated interleukin (IL-6) synthesis in osteoblasts by CGA (Yamamoto et al., 2015; Kozawa et al., 1997). Osteoblasts are derived from MSCs in the bone marrow, and differentiation is regulated by the bone morphogenetic protein (BMP) and wingless-associated integration site (WNT) pathways, which, in turn, increase cytoplasmic cAMP levels and stimulate DNA and collagen synthesis (Zhao et al., 2018; Ponzetti and Rucci, 2021).

### 2.2 The antioxidant and anti-apoptotic effects of CGA

Osteoblast precursors are recruited from the bone marrow to the bone surface by molecules, such as transforming growth factor β (TGF-β) and IGF-14. The first step in differentiation is the activation of runt-related transcription factor 2 (RUNX2), osterix and distal *Drosophila* 5. At the molecular level, RUNX2 is the main transcription factor regulating osteoblast function, while loss-of-function mutations in nuclear factor E2 p45-related factor 2 (Nrf2) reduce bone mass and load-driven anabolic responses (Dirckx et al., 2019; Komori, 2011). The role of Nrf2 in osteoblast differentiation may be related to intracellular reactive oxygen species (ROS) levels, which are elevated in Nrf2-deficient stromal cells, thereby causing oxidative stress and inhibiting osteoblast differentiation (Han et al., 2019; Park et al., 2014). Runx2 also promotes the proliferation of osteoblast progenitors and drives their commitment to the osteoblast lineage by directly regulating the expression of genes involved in hedgehog, fibroblast growth factor (Fgf), Wnt, and parathyroid hormone-like hormone (Pthlh) signaling pathways, as well as distal-less homeobox 5(Dlx5). In addition, smad ubiquitination regulatory factors (Smurfs) negatively modulate the TGF-β/BMP signaling pathway by promoting the ubiquitination and subsequent degradation of key signaling components (Kushioka et al., 2020; Komori, 2022). CGA inhibits apoptosis by reversing increased intracellular ROS production, H_2_O_2_ concentrations, and mitochondrial superoxide overproduction. The decreased expression of RUNX2, a transcription factor important for osteoblast differentiation, led to decreases in the expression of the important extracellular matrix protein osteocalcin. CGA extracts alleviated glucocorticoid-induced increases in bone resorption markers and decreased osteogenic markers by upregulating RANKL/OPG and RUNX2 signaling mRNA expression and protein levels (Naveed et al., 2018; Mo et al., 2019; Cai et al., 2021; Xiao et al., 2020). Zhou *et al.* found that Shp2 is a major cytoplasmic tyrosine phosphatase and that CGA promotes osteoblast proliferation and osteoclast differentiation in Bone marrow mesenchymal stem cells via the Shp2/PI3K/Akt/cyclinD1 pathway (Zhou et al., 2016). Upregulation of cellular proliferation by CGA was blocked by inhibiting Akt or Shp2. Recently, Bax and Bcl-2 were found to be two important genes regulating apoptosis. Bax overexpression accelerates apoptosis, whereas Bcl-2 overexpression inhibits apoptosis. High doses of CGA can prevent decreases in Bcl-2 and increases in Bax during apoptosis, thus inhibiting apoptosis (Zhang and Hu, 2016).

### 2.3 The benefits of CGA on the skeletal system (related to hormones)

One study found that CGA not only ameliorated ovariectomy-induced decreases in bone mineral density, but also upregulated osteocalcin and deoxypyridine, and stimulated alkaline phosphatase activity in primary osteoblasts. In addition, it prevented decreases in osteoblast viability after H_2_O_2_ treatment and reduced the rate of apoptosis and Caspase-3 activity. CGA activates Akt phosphorylation in osteoblasts and protects osteoblasts from oxidative damage via the PI3K/Akt-mediated Nrf2/HO-1 pathway (Han et al., 2019; Han et al., 2017; Nugraha et al., 2023). CGA was reported to reverse the dexamethasone-induced downregulation of p21 and promote the expression of nuclear Nrf2 and total Nrf2, as well as their downstream target protein heme oxygenase-1 (HO-1). These results suggest that the protective effects of CGA in osteoblasts are closely related to the activation of the PI3K/Akt-Nrf2/HO-1 and p21-Nrf2/HO-1 anti-oxidative stress signaling pathways, providing experimental evidence for the cytoprotective effects of CGA (Han et al., 2019; Han et al., 2017). Zhou *et al.* suggested that CGA might inhibit bone resorption in a manner similar to that of phytoestrogens, i.e., by competing with estrogen for estrogen receptors (ERs). In rodents and humans, ERs exist in both α and β isoforms. The Erβ isoforms are more abundant, and CGA has a higher affinity for ERβ. Studies have shown that CGA has a direct stimulatory effect on the proliferation and differentiation of cultured rat osteoblast precursors (Dirckx et al., 2019; Innocenti et al., 2007; Yang and Wan, 2019). At the cellular level, high doses of CGA at a concentration of 20 mg/kg enhanced bone density in the femoral head and femoral neck after ischemia. CGA can improve the proliferative capacity of osteoblasts, accelerate the transition of osteoblasts from the G1 phase to the S phase, and enhance mitosis and osteoblast regeneration, thus effectively improving hormone-induced necrosis (Zhang and Hu, 2016).

### 2.4 Derivatives of CGA and their osteogenic effects

**FIGURE 5:**
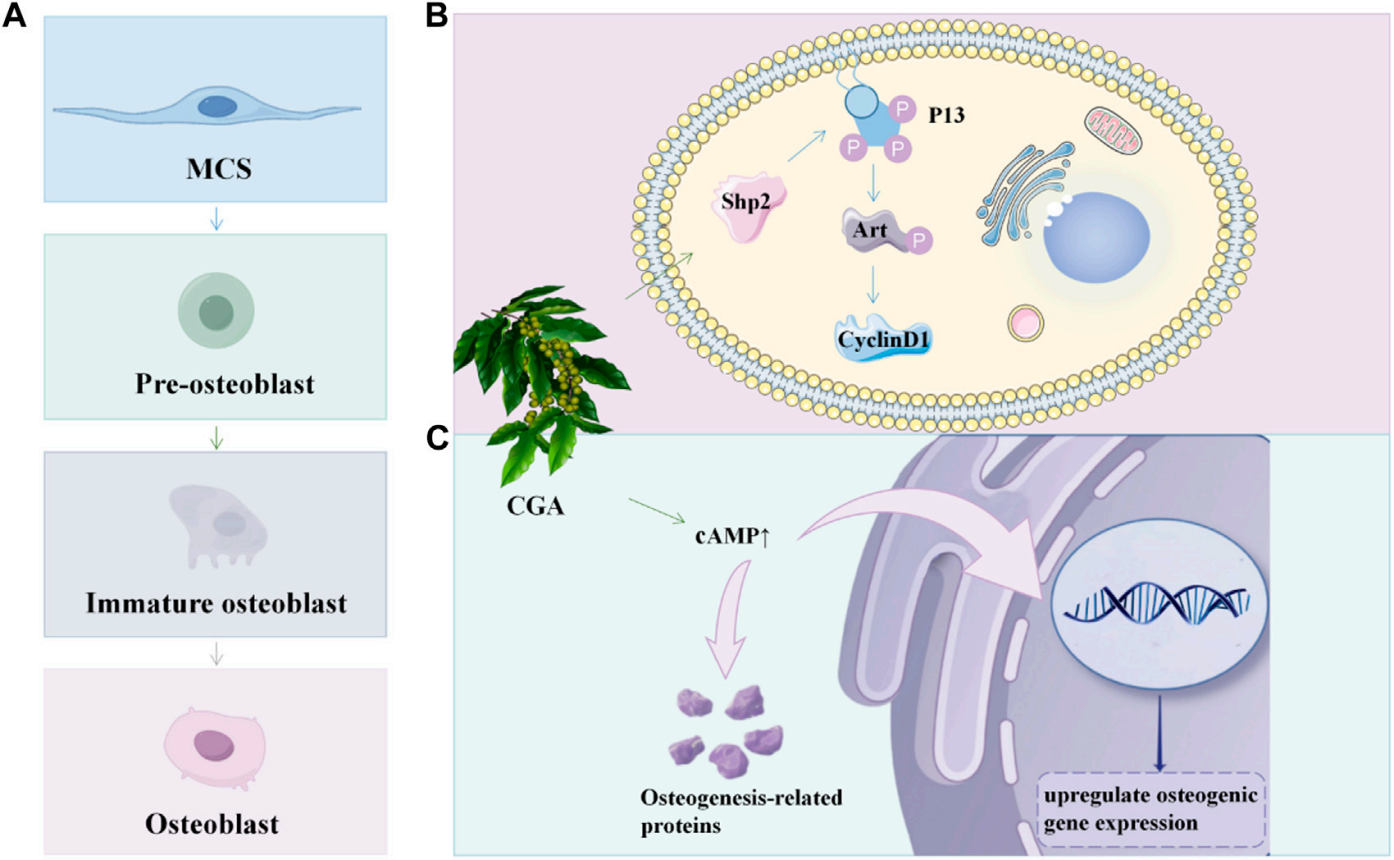
CGA promotes osteoblast proliferation and differentiation **(A)** The developmental process of osteoblasts; **(B)** CGA promote osteoblast proliferation in BMSC via the Shp2/PI3K/Akt/cyclin D1 pathway; **(C)** CGA could upregulate osteogenic gene expression and osteogenic protein synthesis by promoting cAMP expression.

Karadeniz *et al.* found that 3, 5-dicaffeoyl-epi-quinic acid (DCEQA), a derivative of CGA, enhanced osteoblast differentiation by stimulating Wnt/BMP signaling. It upregulated the expression of osteogenic markers alkaline phosphatase, osteocalcin, Runx2, BMP2, and *Wnt10a* (Karadeniz et al., 2020). Min *et al.* found that the *Hspa1a/Fgfr2/Gadd45a/Tgfb3/Hspa1b* gene was localized to the MAPK pathway using Kyoto Encyclopedia of Genes and Genomes analysis. Previous studies reported that CGA might affect the expression of apoptosis-related genes that are part of the oxidative stress and p38 MAP-dependent pathways (Min et al., 2018). (Figure 5) A clinical study conducted by Ferrantelli et al. demonstrated that daily consumption of 100 g of fresh lettuce with high concentrations of polyphenols, including CGA and CA, for a duration of 12 days resulted in the regulation of bone metabolism and improved phosphate absorption rate in participants. Consequently, natural polyphenols exert a positive influence on bone health (Ferrantelli et al., 2023).

However, some scholars remain skeptical of osteogenic promotion by CGA. Sakai suggested that the enhancement of PGF2α-induced Osteoprotegerin mRNA expression levels may not be attributed to CGA but to Epigallocatechin Gallate (Sakai et al., 2017). Folwarczna and colleagues also found that low doses of caffeic acid had detrimental effects on the skeletal system, while high doses of caffeic acid and CGA increased tibial mineralization and improved the mechanical properties of the femoral shaft (Folwarczna et al., 2015). In summary, CGA positively influences the proliferation, differentiation, and apoptosis of osteoblasts through various mechanisms, thereby helping to maintain skeletal health. These findings provide experimental evidence and a theoretical basis for applying CGA to treat skeletal diseases, offering potential new drug candidates or therapeutic strategies for the prevention and treatment of related conditions. However, further research is needed to explore the specific mechanisms of action of CGA *in vivo*, as well as its safety and efficacy in different populations and disease models.

## 3 Chlorogenic acid inhibits osteoclasts

### 3.1 The interaction between osteoclasts and osteoblasts

Osteoclasts are derived from mononuclear hematopoietic marrow lineage cells and are attracted to the bloodstream and then to the bone surface by factors such as sphinxin-1 phosphate. Multinucleated bone-resorbing cells are formed by the uptake of chemokines and other factors. The interaction between osteoblasts and osteoclasts is crucial for maintaining site-specific bone homeostasis and regulating bone remodeling. Both small extracellular vesicles and apoptotic vesicles from mature osteoblasts were shown to express membrane-bound RANK, which is a ligand for osteoblastic RANKL-activated RUNX2 expression. Faqeer *et al.* identified a new method of communication between osteoclasts and osteoblasts through proteomics analysis. Specifically, Secreted phosphoprotein one from osteoclasts activates TGFβ1/SMAD signaling in MSCs, promoting their osteogenic differentiation (Faqeer et al., 2023).

### 3.2 The regulatory role of CGA on osteoclasts

#### 3.2.1 CGA regulated the RANKL signaling pathway

In recent years, CGA has been reported to reduce osteoclast differentiation and bone resorption. It was also shown to increase tibial mineralization and improve bone strength of the femoral backbone in *in vivo* experiments, which may be related to its regulation of the RANKL signaling pathway (Kuroyanagi et al., 2017; Chen et al., 2021; Ho et al., 2024). RANKL can bind to the RANK receptor on the surface of osteoblasts, thereby activating a number of major intracellular signaling pathways, including nuclear factor-κB (NF-κB), JNK, ERK, and p38 MAPK. NF-κβ is translocated to the nucleus through IκB kinase (IKK) phosphorylation and degradation. Recently, CGA was recently discovered to block RANKL-mediated IκB-α phosphorylation and degradation, hence decreasing NF-κB activation. In addition, RANKL-induced MAPK kinase activation further leads to the activation of the NFATc1 factor. NFATc1 is thought to be the master transcription factor regulating the terminal differentiation of osteoblasts (Boyce, 2013; Kwak et al., 2013; Ono et al., 2020; Ono and Nakashima, 2018). CGA showed an inhibitory effect on osteoclast formation by downregulating the expression of NFATc1 and also regulated the expression of the osteoclast-specific genes *OSCAR* and *TRAP*. This effectively prevented RANKL-induced osteoclast production and osteolytic bone destruction *in vivo* (Kwak et al., 2013; Ono et al., 2020; Ono and Nakashima, 2018; Tang et al., 2006; Lee S. H. et al., 2017; Xu et al., 2022). The RANKL signaling pathway plays an important role in bone resorption, and factors affecting osteoclasts all act directly or indirectly through this pathway. Osteoclasts secrete OPG, which acts as a decoy receptor to bind to RANK ligands and prevents them from binding to RANK on osteoclasts, thereby inhibiting bone resorption (Faqeer et al., 2023; Simonet et al., 1997). Osteoclast development may be regulated indirectly through the expression of OPG and RANKL in osteoblasts. The binding of RANKL to receptors is induced by low levels of ROS and multiple growth factors and cytokines (Lee et al., 2005; Ashtar et al., 2020). Hence, Increased ROS may play an important role as a secondary messenger in the RANKL-mediated osteoclast differentiation signaling pathway.

#### 3.2.2 CGA regulates V-ATPase and autophagy

**FIGURE 6:**
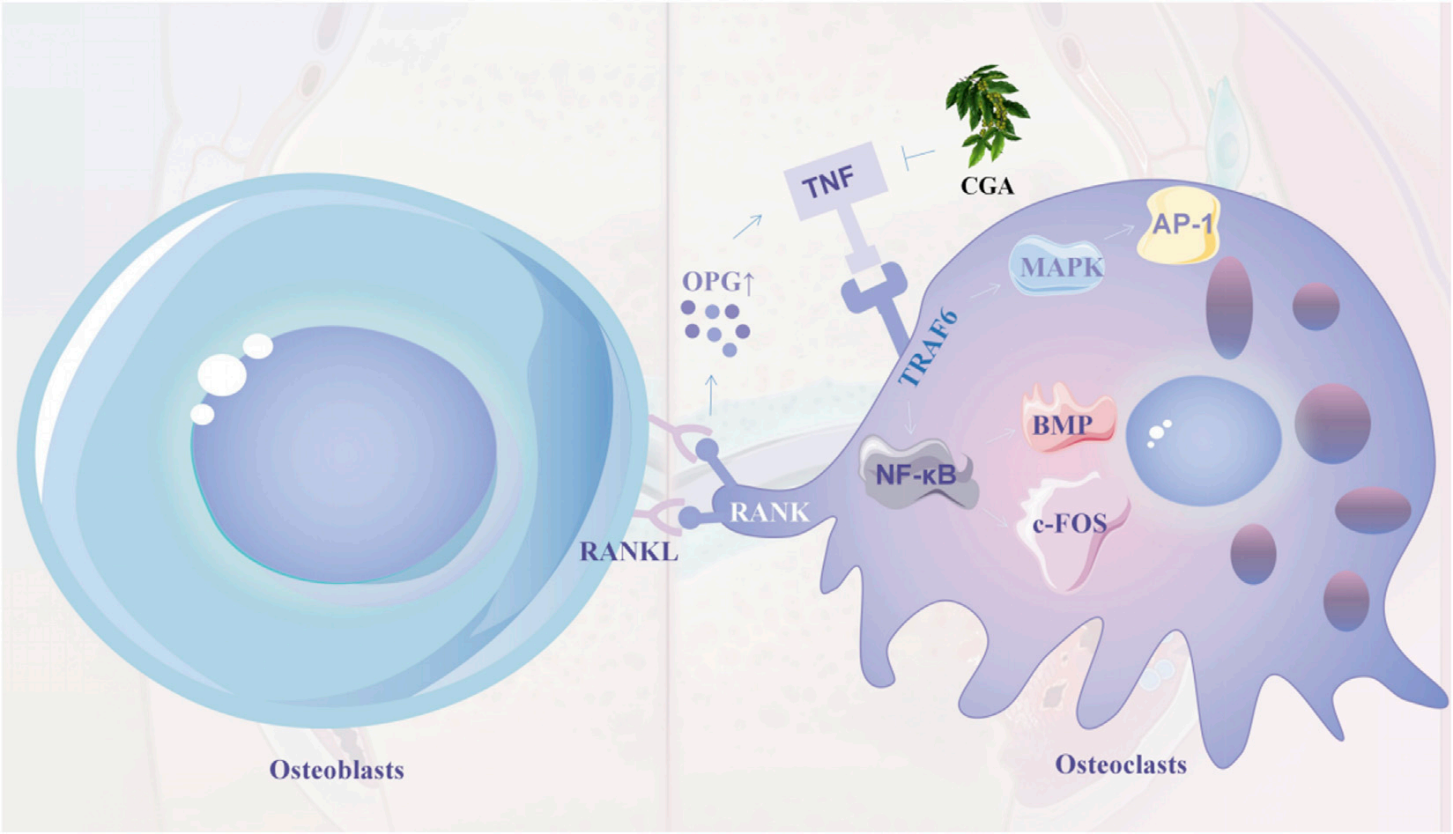
OPG is upregulated after RANKL and RANK junction, thereby recruiting TRAF6 molecules and activating MAPK and NF-κB pathways, thereby promoting the expression of AP-1, BMP and c-FOS (early signals in endochondral bone formation). Chlorogenic acid inhibits osteoclast formation and differentiation by inhibiting TRAF6 and MAPK and NF-κB signalling pathways in osteoblasts. 7.

Emerging evidence suggests that bone resorption requires osteoclasts to possess the capability to generate protons, as the dissolution of the bone matrix by alkaline salts and the digestion of the bone matrix by acid phosphatases secreted by osteoclasts necessitate an acidic pH. The ruffled border of osteoclasts contains vacuolar H + -adenosine triphosphatase (V-ATPase), which hydrolyzes ATP to produce protons that are then translocated to the extracellular environment. The interaction of V-ATPase with TNF receptor-associated factor 6 (TRAF6), which is recruited by RANK, leads to its activation. Lee *et al.* found that CGA, while not significantly affecting the expression of TRAF6 or V-ATPase, blocked the association between V-ATPase and TRAF6 to some extentt (Rousselle and Heymann, 2002; Lee S. H. et al., 2017; Yang Y. et al., 2022). (Figure 6) A recent study found that the processes of endocytosis, secretion, and translocation, which occur at the fold boundary, may be related to cellular autophagy. The loss of lysosome-microtubule connections impairs bone resorption and lysosomal distribution in osteoblasts. The formation of fold boundaries requires several autophagy-associated proteins (Atg) to act in concert with microtubule-associated protein light chain three for actin ring formation, tissue proteinase K release, and bone resorption in osteoclasts (Pantoom et al., 2021; Fujiwara et al., 2016; Hiura et al., 2022; Wang et al., 2011). Caffeic acid, which is structurally similar to CGA, was found to effectively inhibit the formation of the osteoclast differentiation factor NF-κB ligand (receptor activator of RANKL). It directly inhibits osteoclast differentiation by inhibiting NF-κB activity and downregulating c-Fos and NFATc1. Dried plums rich in caffeic acid and CGA induced reductions in osteoclast surface areas, inhibited intrinsic osteoclast activity (reduced ctsk expression), and decreased serum C-terminal cross-linking telopeptide levels (a marker of bone resorption) (Ha et al., 2009; Shahnazari et al., 2016).

Overall, CGA can inhibit osteoclasts by regulating the RANKL signaling pathway, V-ATPase, and autophagy. Therefore, CGA can be used to provide new strategies and methods for the prevention and treatment of bone-related diseases.

## 4 Chlorogenic acid in clinical applications

### 4.1 Applications in the oral cavity

**FIGURE 7:**
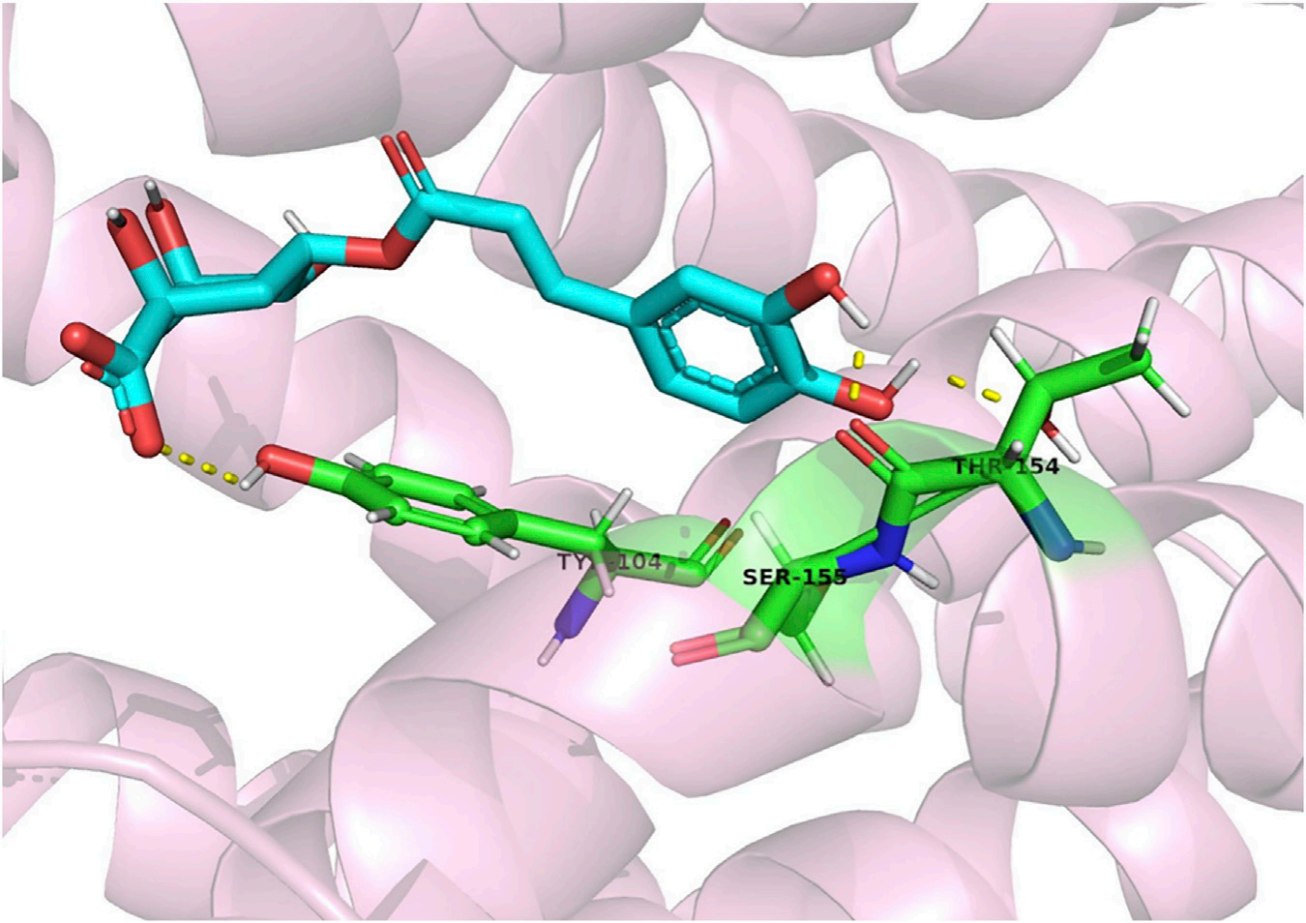
CGA forms a molecular docking with CysLT1R, which in turn reduces lipopolysaccharide-induced inflammation in gingival fibroblasts by affecting the CysLT1R/Nrf2/NLRP3 signalling pathway (Huang et al., 2022).

Periodontitis is a common oral disease affected by dental plaque. Xia *et al.* found that CGA modulates the PI3K/AKT and NF-κB/MAPK signaling pathways targeting key genes (AKT, MAPK1, MAPK14, NF-κB, TNF, IL-2, and IL-1B). CGA treatment also inhibited *Porphyromonas gingivalis* (PG)-lipopolysaccharide (LPS)-induced increases in IL-1β and IL-18 in gingival fibroblasts. It also inhibited the expression of CysLT1R, HO-1, NLRP3, ASC, cysteinogen-1, active cysteine-1, and other proteins associated with LPS induction. In conclusion, CGA inhibited oxidative stress by promoting the nuclear translocation of Nrf2, and increased mitochondrial membrane potential, thereby decreasing the protease activity of PG, reducing alveolar bone loss, and inhibiting the development of inflammation (Kwon et al., 2021; Xia et al., 2023; Scannapieco and Gershovich, 2020; Huang et al., 2022; Tsou et al., 2019). Some authors found that CGA treatment significantly inhibited the production of inflammatory mediators and the expression of inducible nitric oxide synthase (iNOS) and cyclooxygenase-2 (COX-2) proteins mediated by PG in a dose-dependent manner by the Griess reaction, enzyme-linked immunosorbent assay (ELISA), and Western blotting analysis. Treatment of cells with the indicated concentrations of CGA and PG-LPS (1 μg/mL) at 37°C for 4 h revealed that CGA treatment inhibited the activation of the Toll-like receptor 4/myeloid differentiation primary response gene 88 (MyD88) and NF-κB in LPS-PG-stimulated HGFs (Human gingival fibroblasts). CGA treatment also inhibited LPS-PG-induced ERK and Akt phosphorylation but did not affect JNK or p38. The inhibitory effect of CGA on PG was investigated by turbidity tests and plate counts. The minimum inhibitory concentration of CGA was found to be 4 mg/mL, and the minimum bactericidal concentration was found to be 16 mg/mL (Naveed et al., 2018; Tsou et al., 2019; Park and Yoon, 2022; Marchesan et al., 2018; Liang and Kitts, 2015; Rashidi et al., 2022; Arfian et al., 2019). A concentration of 4 mg/mL CGA reduced cysteine protease activity by more than 40%. Hu *et al.* treated human dental pulp stem cells with CGA for 72 h. Cell proliferation in the 100 μg/mL group was significantly increased compared to the 0.1, 10 μg/mL, and 1 mg/mL groups. Frizzled-elated protein (FRZB), a member of the secreted frizzled-related family proteins, plays an important role in the osteogenic differentiation of MSCs as a regulator of Wnt signaling. RNA sequencing and real-time quantitative polymerase chain reaction analyses showed that CGA treatment increased the production of FRZB and pyruvate dehydrogenase kinase 4(PDK4) osteogenic gene expression and suppressed that of asperulins and cytokine-like 1. Western blot analysis revealed that in addition to FRZB, CGA treatment decrease total β-linked protein activity and increased total calcium/calmodulin-dependent kinase II, phosphorylated CamKII, and phosphorylated cAMP response element-binding protein (Hu et al., 2021; Yagi et al., 2022; Corr and Lane, 2007; Thysen et al., 2016). CGA-PLGA was recently synthesized by loading CGA onto poly (D,L-propylene-co-ethyleneglycolate) (PLGA) and modifying it with polyvinylpyrrolidone, resulting in the sustained release of CGA, which not only effectively removed ROS but also inhibited the cellular overexpression of pro-inflammatory cytokines. CGA-PLGA remained in gingival tissue for more than 24 h after local injection in a mouse model, thus effectively inhibiting alveolar bone resorption and stopping the progression of periodontitis (Li et al., 2022). In conclusion, CGA alleviates inflammation and oxidative stress induced by PG by modulating signaling pathways and gene expression, thereby reducing alveolar bone loss. CGA also inhibits bacterial growth and biofilm formation. The development of CGA nanocarriers has facilitated the sustained release of CGA in gingival tissue, suppressing disease progression and offering a novel strategy for treating periodontitis. Palaniraj *et al.* developed an antimicrobial CGA-loaded porous nanogel based on a calcium phosphate-chitosan nanogel. The base nanostructure was formed by the ionic gelation of calcium phosphate nanoparticles as a cross-linking agent, and its porous structure enabled controlled drug release. The negative charge on the phosphate ion and the positive charge on chitosan neutralize bacterial growth. Chitosan and CGA can disrupt bacterial cell membranes and penetrate the cells, thus eliminating biofilm formation. Future nanogel designs could promote the differentiation of human dental pulp MSCs (Palaniraj et al., 2019). He *et al.* addressed the problem of massive defects and associated infections in clinical bone implants by loading a CGA drug/graft peptide (bone-forming peptide, BFP) hydrogel system onto the surface of sulfonated polyetheretherketone (SPEEK). Sodium alginate was used as the drug carrier, and the BFP peptide was grafted onto the sodium alginate surface by further modification. The results showed that the SPEEK-CGA-grafted peptide hydrogel system was more active than CGA in terms of cell adhesion, proliferation, and ALP activity, thus providing an alternative approach for bone repair (Chahardoli et al., 2023; He et al., 2019). (Figure 7)

### 4.2 Applications in other diseases

CGA, renowned for its notable antioxidant and anti-inflammatory properties, mitigates oxidative stress and inflammatory responses in chondrocytes through diverse mechanisms. CGA suppresses the proliferation of osteosarcoma cells and demonstrates potential in treating osteoarthritis and rheumatoid arthritis. CGA also regulates the immune system by reducing the expression of pro-inflammatory cytokines and modulates the balance between bone resorption and formation in osteoporosis. Nanocarriers and hydrogel systems loaded with CGA offer novel therapeutic strategies for treating related skeletal diseases (Yang et al., 2023). The antioxidant potential of CGA has been suggested as it can prevent oxidative stress by activating multiple signaling pathways in chondrocytes. First, CGA can inhibit the STAT3 snail pathway, thereby reducing cell viability and decreasing the expression of the proliferating cell nuclear antigen (PCNA) in osteosarcoma cells. CGA has also been shown to have anticancer effects in several types of tumors, including osteosarcoma. Researchers reported changes in the expression of the signal transducer and activator of transcription 3 (STAT3)/snail pathway by Western blotting analysis to argue the toxicity of CGA on osteosarcoma cells and explore its potential mechanism. Zhang *et al.* found that CGA dose-dependently decreased cell viability and PCNA expression in osteosarcoma cells. At the same time, CGA increased apoptosis and cysteine-3/7 activity in osteosarcoma cells in a concentration-dependent manner. Si-TAT3 inhibited the STAT3/snail pathway to retard the growth of osteosarcoma cells and induce apoptosis. Studies have shown that the induction of apoptosis is the main way in which CGA affects the cell cycle and inhibits cell growth. On this basis, the cooperative effects of CGA and adriamycin were investigated in U2OS and MG-63 human OS cells. In cells treated with adriamycin, the concomitant administration of CGA further reduced cell viability and growth and potentially promoted cell death by inducing apoptosis (Zhang et al., 2019; Sapio et al., 2020; Salzillo et al., 2021; Xu et al., 2023). Yang and colleagues reported the preparation of AuNR@CA nanohybrids from CGA and gold nanorods. These nanohybrids triggered apoptosis and inhibited the growth of osteosarcoma. Additionally, under controlled mild near-infrared irradiation, the nanohybrids stimulated cellular osteogenic differentiation by upregulating Hsp47 and Hsp70 genes and promoting the expression of heat shock proteins (Yang et al., 2023).

Osteoarthritis (OA) is a progressive disease of cartilage damage. The pro-inflammatory cytokine IL-1β plays a key role in the progression of OA by inducing inflammation and upregulating the expression of matrix metalloproteinases (MMPs), a major mediator of bone degradation, leading to cartilage matrix degradation. IL-1β also induces the production of the inflammatory mediators nitric oxide (NO) and prostaglandin E2 (PGE). Chen *et al.* studied the IL-1β-induced expression levels of MMP-1, MMP-3, MMP-13, and tissue inhibitor of metalloproteinase-1 (TIMP-1) in rabbit chondrocytes using quantitative real-time fluorescence PCR and ELISA, and found that CGA inhibited MMP mRNA and protein expression and increased TIMP-1 expression at the mRNA and protein levels. CGA inhibited IL-1β-induced NF-κB activation and degradation of the κB inhibitor (IκB)-α. Other investigators examined the expression levels of iNOS, PGE2, COX-2, collagen II, MMP13, NF-κB, and inhibitor-κB α by Western blot analysis. The results showed that CGA prevented IL-1β-induced increases in iNOS/NO, IL-6, MMP-13, and COX-2/PGE2 production, and the IL-1β-mediated downregulation of collagen II. The data also suggest that CGA inhibits IL-1β-induced inflammatory responses associated with the NF-κB signaling pathway. Taken together, CGA has potential value for treating OA (Huh et al., 2012; Zheng et al., 2023; Chen and Wu, 2014; Liu et al., 2017; Zada et al., 2021; Blaney Davidson et al., 2007). Additionally, it downregulates the expression of pro-inflammatory cytokines, such as TNF-α, IL-6, interferon-gamma (IFN-γ), and MMP-associated proteins. CGA was found to prevent the inflammatory responses induced by IL-1β, which are associated with the NF-κB signaling pathway. This suggests that CGA may have potential value in treating OA (Singh et al., 2023).

Rheumatoid arthritis (RA) is a systemic inflammatory disease, and B-cell activating factor (BAFF), a member of the TNF) family, was recently found to play a key role in the pathogenesis and progression of RA (Smolen et al., 2016). CGA was found to reduce the DNA binding activity of NF-κB to the BAFF promoter region and inhibit BAFF via the NF-κB pathway in TNF-α-stimulated MH7A cells, thereby significantly attenuating the progression of arthritis. At a dosage of 40 mg/L, CGA effectively controlled the total CD3 cell counts and significantly inhibited the binding of CD80/86 to T-cell receptor. The interaction of CD80 or CD86 with the T-cell receptor promotes T-cell responses, enhancing the expansion of antigen-specific T cells. Thus, it can be hypothesized that CGA reduces inflammation by inhibiting T cells, possibly by inhibiting CD80 and CD86 molecules (Fu et al., 2019; Chauhan et al., 2012). The effect of CGA on CD4 T-cell-specific Th1/Th2 cytokines was examined. CGA significantly inhibited Th1 cytokines but elevated Th2 cytokines in a dose-dependent manner. The administration of ASEDS, an aqueous seed extract of *Astragalus membranaceus* rich in CGA, markedly improved immune cell counts, immunoglobulin synthesis, high-density lipoproteins concentrations, and antioxidant status in treated rats (*p* < 0.05). A therapeutic effect on arthritic caused by systemic lupus erythematosus (SLE) was also seen. In an SLE model, 10-week-old female MRL/lpr mice were treated with 40 mg/kg CGA daily for 12 weeks by intraperitoneal injection. Scholars observed that CGA significantly reduced joint swelling in the MRL/lpr mice. Pathological examination showed arthritic cell infiltration and cartilage erosion in the model group, and a slight relief of joint lesions was observed in the CGA group. Therefore, CGA is also gradually being used to treat RA (Joshua et al., 2022; Lee et al., 2018). Lee *et al.* examined the effect of CGA on septic arthritis caused by *Candida albicans* and found a significant reduction in edema (Lee et al., 2008).

Osteoporosis is a systemic skeletal disease characterized by reduced bone mineral density and is particularly prevalent among the elderly and women. Postmenopausal osteoporosis is the most common form in women, with approximately 50% experiencing of women at least one fracture after menopause. This condition is typically caused by an imbalance between excessive bone resorption and insufficient bone formation. Estrogen deficiency further impairs the function of osteoblasts, exacerbating bone loss and intensifying the imbalance between bone absorption and formation (Cui et al., 2024; Cui et al., 2022a; Cui et al., 2022b).

Osteoporosis is characterized by reduced bone mass, deteriorating microarchitecture, and fragility fractures, which are often seen in older women. The anti-osteoporotic activity of *Artemisia capillaris* was investigated using an ethanolic extract of capillary salt (ACHE) containing CGA, caffeic acid, gibberellin, isoquercitrin, isochlorogenic acid A, and hyoscovone. ACHE, particularly CGA, reduced osteoclast differentiation and bone resorption. CGA downregulated the interaction of V-ATPase with TRAF6, partly mediating the blockade of bone resorption by capillaries (Lee et al., 2016; Lee S. K. et al., 2017; Lane et al., 2000).

Finally, CGA/BFP hydrogel systems loaded onto SPEEK surfaces demonstrated excellent antimicrobial activity, offering a potential treatment for defects and associated infections in clinical bone implants (Salzillo et al., 2021; Nandhini et al., 2023). (Table 2)

**TABLE 2:** Chlorogenic acid in clinical applications.

Diseases	Mode	The main mechanism (pathways or key molecules)	The used cells and animals	References
**Periodontitis**	Chlorogenic acid (CA, with 98% purify)	CysLT1R/Nrf2/NLRP3; PI3K/AKT and NF-κB/MAPK	Gingival fibroblasts	Huang et al. (2022)
	A stock solution of chlorogenic acid (128 mg/mL) was prepared in 10% DMSO	Inhibition of Porphyromonas gingivalis growth by chlorogenic acid	Porphyromonas gingivalis	Tsou et al. (2019)
	CGA were dissolved in dimethyl sulfoxide (DMSO)	TLR4/MyD88-mediated PI3K/Akt/NF-κB and MAPK signaling pathways	Human gingival fibroblast	Park and Yoon (2022)
	CGA	Wnt/β-catenin signaling,frizzled-related protein (FRZB) and pyruvate dehydrogenase kinase 4 (PDK4)↑, asporin (ASPN) and cytokine-like 1 (CYTL1)↓	Human dental pulp stem cells	Hu et al. (2021)
	CGA–PLGA@PVP nanomicelles	ROS↓,inflammatory↓	Human gingival fibroblast, Mouse periodontitis model	Li et al. (2022)
**Dental caries**	antimicrobial chlorogenic acid-loaded porous nanogel based on calcium phosphate - chitosan nanogel CaPNP@Chi@CGA	promote odontoblast differentiation of mesenchymal stem cells from human dental pulp	HaCaT cells, Human dental pulp mesenchymal stem cells	Palaniraj et al. (2019)
**Bone restoration**	SPEEK/CGA/BFP	growth factors (BFP) stimulated the proliferation and differentiation of osteoblasts; Gram-positive and Gram-negative resistance	MC3T3 cells	He et al. (2019)
**Osteosarcoma**	CGA	Signal Transducer and Activator of Transcription 3 (STAT3)/snail pathway	Human osteosarcoma cell lines MG-63 and Saos-2	Zhang et al. (2019)
	CGA	Extracellular-signal-regulated kinase1/2 (ERK1/2)	U2OS, Saos-2, and MG-63 OS cells	Sapio et al. (2020)
	CGA + doxorubicin (Doxo)	p44/42 MAPK pathway	U2OS and MG-63 human OS cells	Salzillo et al. (2021)
	AuNR@CA	the Hsp47 and Hsp70 genes and promoting the expression of heat shock proteins	Saos-2 cells,MC3T3-E1 cells, the nude mice bearing Saos-2 tumor	Yang et al. (2023)
	CGA	MMP-1、MMP-3、MMP-13、ADAMTS-4、ADAMTS-5↓, MAPK pathway	Chondrocytes	Huh et al. (2012)
	CGA	NO synthase (iNOS) and cyclooxygenase (COX)-2	Chondrocytes	Chen and Wu (2014)
	CGA	IL-1β; iNOS/NO、IL-6、MMP-13、COX-2/PGE2	SW-1353 chondrocytes	Liu et al. (2017)
	CGA	Autophagy	Human chondrocyte C28/I2 cells	Zada et al. (2021)
**Rheumatoid arthritis**	CGA	NF- κ B Signaling Pathway, BAFF	Human Synoviocyte MH7A Cells	Fu et al. (2019)
	CGA	CD4⁺ T cells specific Th1/Th2 cytokines	CD3, CD4, CD8 T cells	Chauhan et al. (2012)
**Septic arthritis**	CGA	CGA inhibited growth of C. albicans yeast cells	*Candida* albicans, mice with septic arthritis	Lee et al. (2008)
**Skin wound healing**	polyvinyl alcohol hydrogel containing chlorogenic acid microspheres	CGA hydrogel significantly increased epithelialization and production of collagen fibers	NIH3T3 cells	Nandhini et al. (2023)

## 5 Discussion

The current review has several limitations. Firstly, the optimal concentration of CGA for osteogenesis must be investigated, and specific mechanisms by which CGA inhibits osteogenesis at low concentrations while promoting it at high concentrations must be elucidated to lay the groundwork for the future development of CGA-related drugs to enhance bone formation. Secondly, the inconsistency in the dosages and models used in current experimental research poses a challenge in reaching consistent conclusions. Lastly, few studies have investigated the effects of CGA on osteoclasts and osteoarthritis, and clinical trials assessing the safety and efficacy of CGA in diverse populations and disease models are lacking.

## 6 Conclusion

The increasing number of bone-related diseases has helped them to become a pressing concern, especially for the aging population facing challenges associated with such disorders. Oral-related bone issues diminish the quality of life and impose significant financial burdens. Chronic bone illnesses can progress over time, potentially resulting in fractures and disabilities (Chen et al., 2019; Aspray and Hill, 2019; Lorentzon and Cummings, 2015; Parsons et al., 2018; Black and Rosen, 2016; Schwendicke, 2020; Nazir, 2017). Currently, the primary clinical treatment for osteoporosis in Western medicine involves bone resorption-inhibiting drugs, which often have extended treatment cycles and substantial adverse effects. These issues make it challenging for patients to adhere to the treatments over extended periods, resulting in suboptimal outcomes (Prestwood et al., 1995; Miller, 2016; Lei et al., 2023).

In contrast, CGA controls cellular signaling pathways influencing osteoblast and osteoclast development, promotes bone production, prevents bone resorption, and exhibits potent osteogenic characteristics. In recent years, research on senescence and bone diseases has gradually increased. Ambrosi *et al.* reported that senescent cells tended to accumulate in bone and trigger chronic inflammation by releasing secreted phenotypic factors associated with senescence. Several signaling pathways, including Hedgehog, Notch, Wnt/β-linker protein, TGF/BMP, and fibroblast growth factor, are involved in regulating cellular senescence in the bone and bone marrow microenvironments (He et al., 2023; Ambrosi et al., 2021). CGA has been shown to inhibit endothelial senescence both *in vivo* and *in vitro* by regulating the Nrf2/HO-1 pathway (Hada et al., 2020). CGA activates the FOXO transcription factors DAF-16, HSF-1, SKN-1, and HIF-1 and prolongs the lifespan of *Hidradenitis elegans* nematodes through DAF-16 in the insulin/IGF-1 signaling pathway. Therefore, CGA is expected to regulate bone regeneration and treat bone-related diseases through senescence-related pathways (Zheng et al., 2017).

Although few clinical trials have focused on CGA, the existing studies show promising results for the potential of CGS to protect bones. Research outcomes in mice suggest that CGA has the ability to protect various macromolecules against oxidative damage. As a significant source of polyphenols in the human diet, CGA is considered a promising candidate for the treatment of bone-related conditions. Further studies should delve into how CGA interacts with biological processes and its potential application in preventing or treating bone deformities.

The available safety evidence suggests that CGA should be relatively safe for use in enhancing bone health. However, recent studies have indicated that CGA can act as an allergen in certain individuals, potentially triggering allergic reactions such as asthma and dermatitis (Lin et al., 2013). Therefore, dose-specific clinical trials are needed to determine the safety of its long-term use before it can be clinically applied. In recent years, with the advancement of biomedicine, topical drug delivery systems have gained popularity in clinical settings due to their low side effects, high concentrations in target tissues, and low systemic uptake (Zhao et al., 2022). Rui *et al.* found that CGA-chitosan complex significantly enhanced metal ion-chelating activity, total antioxidant capacity, scavenging activity, and lipid peroxidation (Rui et al., 2017). CGA also exhibited excellent antibacterial activity against both Gram-negative and Gram-positive bacteria (Yang X. et al., 2022). Determining the effective dose of CGA to promote osteogenesis will be crucial for future clinical trials and investments. In the dental field, CGA is anticipated to be used to treat periodontitis and peri-implantitis (Zhou et al., 2016; Sari et al., 2023).^.^
